# Teacher performance, attitude and classroom practices dataset collected to evaluate the Rwandan Quality Basic Education project

**DOI:** 10.1016/j.dib.2022.108758

**Published:** 2022-11-18

**Authors:** Pheneas Nkundabakura, Theophile Nsengimana, Celine Byukusenge, Aloys Iyamuremye, Jane Batamuliza, Jean Nepomuscene Twahirwa

**Affiliations:** a University of Rwanda College of Education (URCE), Kayonza, P.O Box 55 Rwamagana, Rwanda; b Rwanda Quality Basic Education for Human Capital Development (RQBEHCD) Project; c African Center of Excellence for Innovative Teaching and Learning Mathematics and Science (ACEITLMS)

**Keywords:** Dataset, Classroom practices, Continuous professional development, Teachers’ performance, Teacher's beliefs, Mathematics and science, Primary and secondary school, Rwanda

## Abstract

The Rwanda Quality Basic Education for Human Capital Development (RQBEHCD) project is financed by Word Bank Group through the Government of Rwanda and was embarked on in 2020. It aimed to provide the necessary support to upper primary and lower secondary school teachers and equip them with enough content knowledge, lab skills, pedagogy, and the use of technology in teaching. The dataset presented here comprises data collected from teachers selected in the ten districts of Rwanda. Data were collected in three ways: (a) teachers’ performance in mathematics and science subjects, (b) teachers' beliefs, and (c) classroom observation. Both quantitative and qualitative approaches were used to collect data. Data were collected before and after delivering Continuous Professional Development (CPD) training on Teacher Pedagogical Content Knowledge and the use of modernized tools and approaches. The data were gathered between July 2021 and June 2022, and teachers received the CPD training from August 2021 to April 2022. Teachers' performance data in mathematics and science subjects were collected from 657 lower secondary and 290 upper primary teachers using the teachers' performance achievement test, while beliefs data were collected from 691 secondary and 290 primary teachers using the attitude test. Furthermore, 659 lower secondary and 300 upper primary Mathematics and Science teachers were observed using the Kobo-toolbox-generated Classroom-Based Monitoring and Evaluation Tool (CMET). This data article explains how we created, collected, and analyzed data for the study. Thus, this dataset is presented in the form of raw and analyzed data. The presented data provide information teachers’ performance and beliefs in Mathematics and Science; it also enables researchers to reanalyze it based on the variables of interest. In addition, it provides a broad overview of the impact of CPD on teaching mathematics and Science in primary and secondary schools to policymakers, educators, researchers, and other stakeholders.

**Specifications Table**

**Table:** 

Subject	Mathematics and Science
Specific subject area	Mathematics and Science Education
Type of data	Table Graph Figure
How the data were acquired	Three types of data were gathered, typically teachers' performance, beliefs, and classroom practices. The first data on teachers' performance and conceptual understanding were gathered using the Mathematics and Science Achievement Test. All tools are available in supplementary materials or at https://njctl.org/online-learning/teachers/. The second type of data was on teachers’ beliefs in mathematics and Science were collected by using the Belief about Reformed Science Teaching and Learning tool (RSTL) adapted from [1]. The third type of data on teaching practices was gathered through the Classroom Observation Monitoring and Evaluation tool (CMET) generated by the KoboTool box available at *(*https://ee.kobotoolbox.org/x/QnDTFdr9*). All data were recorded in Microsoft excel 2016 and SPSS V.25.*
Data format	Raw Analyzed Filtered
Description of data collection	Data on teachers' performance and beliefs were collected from 891 Mathematics and science teachers (146 chemistry, 180 Biology, 148 Physics, 139 Science and Elementary Technology (SET), 151 Mathematics (primary), and 127 Mathematics (secondary). To evaluate teachers’ belief in mathematics and Science, 691 teachers, including 290 primary and 401 secondary teachers, responded an aptitude test. In addition, the 959 teachers, specifically the 300 primary and 659 lower secondary teachers, were observed to evaluate how well they used the resources provided and the innovative teaching techniques after training received from August 2021 to April 2022.
Data source location	District: 10 districts (Rustiro, Gakenke, Nyagatare, Rulindo, Gatsibo, Muhanga, Huye, Bugesera, Kirehe, and Nyarugenge). Country: Rwanda Sample: 959 upper primary and lower secondary mathematics and science teachers.
Data accessibility	Data are available freely to explore and reuse Repository name: Mendeley Data identification number: 10.17632/6xv7z8rh54.1 Direct URL to data: (https://data.mendeley.com/datasets/6xv7z8rh54/1

## Value of the Data

• The presented data are valuable and beneficial to mathematics and science education in Rwanda as they elucidate the status of teachers’ content knowledge and their perceptions about teaching those subjects.
• These data are of great importance because they provide insights into the usage of modernized tools and innovative teaching and learning methods in mathematics and science subjects at the upper primary and lower secondary Rwandan classrooms.
• The data about teachers’ performance in mathematics and sciences before and after Continuous professional training are of great importance for further educational development initiatives.
• Policymakers and Curriculum designers can do relevant reviews that reflect competence among teachers, advocate for relevant, innovative teaching methodologies and effective implementation of a Competence-Based Curriculum, and determine professional development needs for the teachers.
• These data can be re-used by researchers in similar fields to measure the effect of CPD training on teachers’ professional performance and identify gaps and predict possible remedies. Thus, data can be analyzed into various variables such as School location, type, and ownership, district, Gender, subject, teachers’ experience, and qualification.

## 1 Objective

This dataset intends to evaluate the impact of CPD training on Rwandan teachers across upper primary and lower secondary schools. The following are the objectives:

❖ To assess the impact of CPD training on teachers’ performance and beliefs in mathematics and science teachers in the upper primary and lower secondary schools in Rwanda.
❖ To evaluate how mathematics and science teachers apply modernized tools within the upper primary and lower secondary schools.
❖ To compare the use of modernized tools and innovative methods of teaching and learning mathematics and Science based on gender, qualification, district, school ownership, type, and location.

## 2 Data Description

To evaluate the impact of CPD training on teachers' performance and raise the standard of Mathematics and Science teaching and learning, we gathered nine datasets. Each dataset exists in a single Microsoft Excel 2016 file. Three groups were created from the dataset. Data from the first dataset pertains to teachers' performance in math and Science, the second dataset to teachers' attitudes toward math and Science, and the third dataset to classroom observations.

### 2.1 Data set related to teachers’ performance in mathematics and Science

The six files containing the aforementioned data are quite similar to one another; they all contain datasets of teachers' performance in biology, physics, chemistry, Science and elementary technology (SET), mathematics (primary), and mathematics (secondary), which were each composed of seven sheets. The first and second sheet of all file files contains raw data of pre-and post-test scores, respectively. From sheet-3 up to sheet 7 contains analyzed data in the form of figures from sheet-one and two. In every file pertaining to a teacher's performance in math and science, sheets one and two from cells A to S are identical and contain the teacher's identification. However, the differences between columns-T and DW, which represent the questions that were asked and their marking, are the result of the different numbers of questions that were asked for each subject. In all six files, Sheet number three, named “analysis-1” contains three figures of histogram indicating marks distribution in both pre-and post-test, male and females. The average of both pre-and post-test was copied to analysis-1 from sheet one and sheet two. Analysis-1 presents overall pre-and post-test, male and female scores. The figure1,2,3 were captured using the taken range interval and overall pre-and post-test, male, and female scores. Sheet number four, called “analysis-2” comprises two figures demonstrating teachers' performance per question in the pre-and post-test. The total number of questions that were correctly answered and the percentage with regard to gender were calculated in sheets one and two. Following that, copies of each of these data were made and included in analysis-2. The extent to which each question was answered generally in the pre-and post-test as well as by gender, was then depicted in figures.

Sheet five baptized, “analysis-3” encompasses three figures indicating teachers performance in the pre-and post-test per gender. Data in analysis-3 were copied from analysis 2. It comprises the number of questions and how each has been performed in pre-and post-test. Thereafter, questions and post-test scores were fixed and sorted pre-test to generate figures which display how each question has been performed in overall pre, post-test, and gender. Sheet-6 termed “analysis-4” comprises three figures showing teachers’ performance per topic areas in the pre-and post-test, females' and males' performance in the pre and post-test per topic area, and questions answered correctly and incorrectly in the pre and post-test. We copied the data from analysis-2 into analysis 4 for analysis 4. Then, we divided the questions into topic areas and examined how well each topic performed in the pre-and post-test, as well as gender. Following that, the figures were generated to show the extents to which the teachers performed the topics per question. In addition, a figure depicting the ratios of correctly answered and incorrectly answered questions has been created (see analysis-4).

Lastly, sheet-7 named “analysis-5” comprises two figures displaying individual teachers’ performance in the pre-and post-test. We copied the data from analysis-1 into analysis 5 for that analysis. After that, we sorted the pre-test and fixed the individual teacher and post-test. Figures showing the improvement in each teacher's performance from the pre- to post-test, as well as gender, were then taken into account.

### 2.2 Data related to the teachers’ belief in mathematics and Science

The dataset related to teachers’ belief in Mathematics and Science is classified into two files. One file contains data on primary teachers' beliefs while another contains secondary teacher beliefs. The file containing primary teachers beliefs in mathematics and Science is composed by four sheets. Sheet-one, named” MATH-SET ALL TOGETHER” contains participant identification, pre-, and post-attitude test. From the column A to E represents teachers' information(number, teacher name, gender, province, district, subject, qualification, and teaching experience. While column pre- to post-test questions differ depending on the numbers of items used during analysis. The information in column B shows the specific subjects of teachers they taught, whereby from column 4 to 154 represent Mathematics while from raw 155 to 293 contain SET subjects. Sheet number two termed “Analysis-1” comprises a descriptive analysis of pre-and post-test. To simplify analysis-1, the “count if” function and abbreviations were used to determine the extent or percentages to which teachers agree or disagree with the used questions under investigation. D1 stands for items 1 up to 8, D2 stands for items nine up to 16, D3 stands for items 17 up to 24, and D4 stands for items 25 up to 32. Following that, disagree, and strongly disagree were combined to form “disagreement,” while agree and strongly agree formed “agreement.” Analysis one includes the percentage level of disagreement, agreements, and genders.Sheet number three, named “analysis-2” encompasses the analysis of how teacher argues different statements in the pre-and post-test per item. For analysis-2, we copied analysis-1 into analysis 2. To produce the figure, we calculated the difference from post to pre-test. This was done with the aim of checking the level of improvement made by the teachers after receiving the training. Afterward, we produced the figure depicting the level of agreement based on item per item. Additionally, Sheet four, termed “analysis-3” demonstrates the analysis of the level of agreement and disagreement of different statements in the pre and post-test. Similarly, analysis-1 was copied into 3. Thereafter, we separated the level of agreement or disagreement in both pre-and post-test. Subsequently, the figure showing the overall agreement and disagreement across pre-and post-test has been produced

On the other side, file two demonstrates secondary teachers’ belief in mathematics and Science. It contains eight sheets. Sheet number one, named “Raw data secondary all subject,” shows teachers identification and pre-and post-test items. Teachers identification are presented from column A to O, followed with a pre-and post-test item from column P to CJ. Sheet number two, called “CODED NEGATIVELY-POSITIVELY,” shows the statements which was changed from negative to positively statements; it can be shown in the blue color column. Sheet number three, termed “Analysis-1” demonstrate descriptive analysis of pre-and post-test. The information in analysis-1 was copied from the sheet one where “COUNTIF” function was used to compute levels of agreement and disagreement. During computation, disagree, and strongly disagree were combined to form disagreement while agree and strongly agree formed agreement. The sheet number four called “Analysis-2” represent descriptive analysis in terms of gender. Data copied from sheet one was computed based the gender; in this sheet, the level of agreement and disagreement was calculated in terms of percentage. Sheet number five, termed “analysis-3” displays a descriptive analysis of pre-and post-test per item. The data in this sheet analysis-3 were copied from sheet one, in this sheet, we computed analysis of the level of agreement and disagreement item per item, where we calculated the difference between the post-test and the pre-test to create the graph. This was done to assess the level of improvement made by the teachers after receiving the training. Following that, we created a graph depicting the level of agreement item by item. Sheet number six, so-called” analysis-4” contain the figure that display the analysis of teachers beliefs in the pre-and post-test item per item with change. Sheet number seven, named “analysis-5” represents the analyzed figure on the level of agreement and disagreement in the pre and post-test. This file ends with Sheet number eight, called “Analysis 6” which is the figure analysis of levels of agreement and disagreement of items in the pre-and post-test. In this line, the level of agreement and disagreement of each item in the pre-and post-test.

### 2.3 Data related to the classroom observation

The file for classroom observation data contains four sheet. Sheet number one, named “Raw data of observed teachers,” covers anonymous information about observed teachers and observed items during the observation period. From column-AJ represent teachers’ identification, and the title of the lesson observed is displayed in column K. The Classroom-Based Monitoring and Evaluation Tool (CMET) used was generated from Kobo-toolbox (https://ee.kobotoolbox.org/x/QnDTFdr9) and divided into four parts (A, B, C and D). Part A Start from column L-N represents the evaluation of the use of the provided resources, Part B from column P - AE is related to the pedagogical observation, Part C (column AG)is associated to the use of ICT resources, and part D (column AH) deserved for the observer comments. “COUNTIF” function was used to correct incorrect answers given to each observing criterion by assigning a “1” score to the “Yes” option and a “0” score to the “No.” It also shows the percentage of achieved and non-achieved criteria. Sheet-two, termed “analysis-1” represents the analysis in terms of percentages and figures of all observed teachers in all categories based on the use of the provided resources, lesson planning, assessment, classroom interaction, and student engagement. Sheet-three, named “analysis-2” includes the analysis of all variables such as gender, geographical location, and qualification; during the analysis, the following code was given to the variables. Analysis-two also showed how observation was done, yes given “1” while No given 0 by using the COUNTIF function. Sheet number four, termed “Analysis-3,” demonstrate the code given variable, in column E represents the code given to the district (1= Rutsiro, 2=Gakenke, 3= Nyagatare, 4= Rulindo, 5=Gatsibo, 6= Muhanga, 7= Huye, 8=Bugesera, 9 = Kirehe, 10= Nyarugenge), Column F represents school ownership (1= Public, 2= Government aided), Column G shows code given to the school type (1=Boarding, 2= Day school), column H displays school location (1=Urban, 2=Rural), column I shows gender (1=Male, 2=Female), and column J demonstrates code given to level (1=Primary, 2= Secondary).

## 3 Experimental Design, Materials and Methods

To collect data presented in this dataset [2] intended to assess the impact of CPD pieces of training on teachers' content knowledge, beliefs, and classroom practices in upper primary and lower secondary schools. The population comprises all upper and lower-secondary Mathematics and science teachers from 10 districts in Rwanda. However, a total of 959 mathematics and sciences teachers were drowned as a sample. These teachers were chosen based on their location, school type, ownership, and gender. Thereafter, teachers were pre-tested using Mathematics and science Achievement Tests and BARSTLQ to check their conceptual understandings and belief level. Teachers need a bunch of training on Pedagogical Content Knowledge. After the intervention, teachers underwent a post-test to gauge their level of improvement. Furthermore, Classroom Observation and Evaluation Tools (CMET) were also used during the observation period (after training). The overall performance and beliefs data collection employed a quasi-experimental design [3], while classroom observation used a survey design [4]. Thus, performance and beliefs data were collected before and after training to measure the impact of training, while observation data were collected after the training to document the current classroom practices and measure the implementation of ICT tools provided and pedagogical approaches trained.

Two modules for the training were created along this line. The first module covered e-learning and ICT in the classroom, and the second covered innovative teaching methods. Later, this module was divided into three sections: Pedagogy, Content Knowledge, and laboratory activities. During the pedagogy training, teachers were trained in using the 5Es instructional model and using modern assessment tools such as plickers, voting cards, and show-me boards [5]. Additionally, the project also focused on content knowledge training for Mathematics and Science teachers, with a particular emphasis on content that has been identified as difficult and in which teachers have more misconceptions [6]. Furthermore, teachers were trained in lab experiments related to those units, and they were given extensive practical work [7]. UR-CE Trainers attend preparatory sessions organized by RQBEHCD before facilitating or conducting any training session so that they are well equipped with all necessary tips and techniques to better facilitate the training sessions in a harmonized manner. Then after, UR-CE trainers were also trained on how to develop questionnaires from KoboToolbox. KoboToolBox data was exported to an MS Excel 2016 sheet before analysis.

### 3.1 Method for collecting and analyzing teachers' performance in mathematics and science data

Mathematics and Science achievement tests were adopted. These instruments were picked from the standardized tests available at https://njctl.org/online-learning/teachers/ *and* aligned with the competence-based curriculum currently used in Rwanda*.* However, a few other questions were structured by RQBEHCD project staff in cooperation with UR-CE lecturers to ensure that the designed instruments cover the planned contents in the training manuals. Initially, 50 multiple-choice questions were planned to be used for each subject. Thereafter, the instruments were validated by Ph.D. students from the African Centre of Excellence for Innovative Teaching and Learning Science and Mathematics (ACEITLMS). These students were a good fit for validation because they were experienced in research in the field of Mathematics and Sciences and expert also in the field of mathematics and sciences curriculum. After validation, harmonized instruments were prepared. For SET, we left with 56 questions, 38 in Chemistry, 50 in physics, 44 in Mathematics (secondary), 39 in Biology, 43 in Mathematics (primary). Before the training, the pre-test was given to the teachers to check their pre-requisite knowledge and level of understanding. Later, the RQBEHCD project, in collaboration with staff members from UR-CE, offered training to Mathematics and Science teachers to improve their Subject Content Knowledge and pedagogy from August 2021 to April 2022. After training, all teachers did a post-test on 26^th^ June 2022 to check how the training impacted them.

Data from the Kobo Toolbox were transferred into an MS Excel 2016 sheet prior to data analysis. The transferred data were filtered by corresponding to each teacher's pre-test and post-test responses. The “EXACT” function was used to count the number of correct and incorrect answers each individual participant received by assigning a “1” score to the correct answer and a “0” score to the incorrect answer. The data were analyzed in terms of percentages and presented in figures.

### 3.2 Method for collecting and analyzing teachers’ beliefs in mathematics and science data

The second data is related to teachers' beliefs in mathematics and Science before and after attending CPD training. The aptitude test used was developed by [1]. The attitude test in the form of pre-and post-test was composed of 32 rating scale questions composed of four rating scale points (strongly agree (4), Agree (3), Disagree(2), and Strongly disagree (1). The test had four dimensions, and each dimension had eight items. Secondary school teachers took the pre-test online on September 20, 2021, and primary school teachers took it online on October 30, 2021. On the 26th of June 2022, both teachers took an online post-test. Teachers took 40 min to 2 hours to complete the test. The KoboTool box was used to collect both teacher's performance and belief data. It is a free online tool available https://ee.kobotoolbox.org/x/QnDTFdr9m*.* The data were collected online and transferred to Microsoft Excel 2016.

Before analyzing the data, it was transferred from Kobo Toolbox to an MS Excel 2016 sheet, where each answer choice for each question was recorded. The same software was used to perform data analysis. Data were filtered by comparing each teacher's pre-test and post-test responses. The “COUNTIF” function was used to calculate the frequency of matches that chose the same levels (strongly agree, agree, disagree, and strongly disagree). Strongly agree and agree were combined into a single level called “agreement”, while strongly disagree and disagree were combined into a single level called “Disagreement”. The filtered and count data were analyzed in terms of percentages, histograms, line figures, and bar charts.

### 3.3 Method for collecting and analyzing classroom observation data

The third data to be collected was related to classroom observation practices. The Classroom-Based Monitoring and Evaluation Tool (CMET), developed by RQBEHCD project team members by using Kobo Toolbox available at https://ee.kobotoolbox.org/x/QnDTFdr9*.* The tool was comprised of 21 criteria and divided into four parts, namely parts A, B, C, and D, that intended to assess modernized tools and innovative methods. Part A contained an evaluation of the use of resources provided by the project, such as computers, projectors, and scripted lessons. Part B focused on the evaluation of the lesson plan, assessment, interactions in the classroom, and student engagement. The use of ICT resources was also evaluated in Part C; in this section, the observation was mainly focused on how teachers interact with students while utilizing ICT resources such as simulations, experiments, animations, and videos. Part D was only designed for the observer's comment

Before going to observe trained teachers, the observers were assigned schools and teachers to observe. Then, they informed concerned teachers and communicated with head teachers about the planned activity. During observation, the observer sat behind the students while the teachers taught and filled out the observation tool; once one of the established criteria was met, the observer marked it Yes; otherwise, it marked No. Following classroom observation, teachers were given constructive feedback highlighting areas of strength and improvement. The “COUNTIF” function was used to analyze data. It was used to determine the number of correct and incorrect answers given to each observer by assigning a “1” score to the “Yes” option and a “0” score to the “No” option. The sum of the “Yes” and “No” options were computed. Data were descriptively analyzed in terms of percentages, histogram, and bar charts.

## Ethics Considerations

Our work involved human subjects; we confirm that the work adhered to the appropriate ethical principles. Firstly, Rwanda educational relevant authorities in collaboration with world bank have approved this work (PAD3212) and relevant informed consent was obtained from teachers. Participant data has been fully anonymized, and the platform(s)’ data redistribution policies were complied with. In fact, this training was certificate oriented. Thus, every participant had to register at the University of Rwanda College of Education and be given a registration number. Headteachers also were requested to recommend a teacher who is ready to follow and complete the whole training session. On side of the participating teacher, he/she was asked to sign a commitment letter to complete the whole training.

## CRediT authorship contribution statement

**Pheneas Nkundabakura:** Conceptualization, Methodology, Validation, Writing – review & editing. **Theophile Nsengimana:** Conceptualization, Methodology, Validation, Writing – review & editing. **Celine Byukusenge:** Visualization, Data curation, Software, Writing – original draft. **Aloys Iyamuremye:** Visualization, Data curation, Software, Writing – original draft. **Jane Batamuliza:** Visualization, Data curation, Software, Writing – original draft. **Jean Nepomuscene Twahirwa:** Visualization, Data curation, Software, Writing – original draft.

## Declaration of Competing Interest

The authors declare that they have no known competing financial interests or personal relationships that could have appeared to influence the work reported in this paper.
